# Pulmonary Aerosol Delivery of Let‐7b microRNA Confers a Striking Inhibitory Effect on Lung Carcinogenesis through Targeting the Tumor Immune Microenvironment

**DOI:** 10.1002/advs.202100629

**Published:** 2021-07-08

**Authors:** Qi Zhang, Jing Pan, Donghai Xiong, Yian Wang, Mark Steven Miller, Shizuko Sei, Robert H. Shoemaker, Alberto Izzotti, Ming You

**Affiliations:** ^1^ Center for Disease Prevention Research Medical College of Wisconsin Milwaukee WI 53226 USA; ^2^ Department of Pharmacology and Toxicology Medical College of Wisconsin Milwaukee WI 53226 USA; ^3^ Chemopreventive Agent Development Research Group Division of Cancer Prevention National Cancer Institute Bethesda MD 20892 USA; ^4^ Department of Experimental Medicine University of Genoa Genoa 16132 Italy; ^5^ IRCCS Ospedale Policlinico San Martino Genoa 16132 Italy; ^6^ Present address: Center for Cancer Prevention, Houston Methodist Cancer Center, Houston Methodist Research Institute Houston TX 77030 USA

**Keywords:** immunity, let‐7b, lung cancer, miRNA, pulmonary aerosol delivery, single‐cell RNA sequencing

## Abstract

MicroRNAs are potential candidates for lung cancer prevention and therapy. A major limitation is the lack of an efficient delivery system to directly deliver miRNA to cancer cells while limiting systemic exposure. The delivery of miRNA via inhalation is a potential strategy for lung cancer prevention in high‐risk individuals. In this study, the authors investigate the efficacy of aerosolized let‐7b miRNA treatment in lung cancer prevention. Let‐7b shows significant inhibition of B[a]P‐induced lung adenoma with no detectable side effects. Single‐cell RNA sequencing of tumor‐infiltrating T cells from primary tumors reveals that Let‐7b post‐transcriptionally suppresses PD‐L1 and PD‐1 expression in the tumor microenvironment, suggesting that let‐7b miRNAs may promote antitumor immunity in vivo. Let‐7b treatment decreases the expression of PD‐1 in CD8+ T cells and reduces PD‐L1 expression in lung tumor cells. The results suggest that this aerosolized let‐7b mimic is a promising approach for lung cancer prevention, and that the in vivo tumor inhibitory effects of let‐7b are mediated, at least in part, by immune‐promoting effects via downregulating PD‐L1 in tumors and/or PD‐1 on CD8+ T cells. These changes potentiate antitumor CD8+ T cell immune responses, and ultimately lead to tumor inhibition.

## Graphical abstract

The geometric median diameter of let‐7b miRNA is 29.0 nm. Ex vivo images show that the miRNA nanoparticles specifically accumulate in the lungs, with little or no accumulation in other tissues. Let‐7b leads to increased frequency of tumor infiltrating CD8+ T cells, and shifts subsets of CD8+ tumor‐infiltrating lymphocytes (TILs) toward antitumor immunity. Combining aerosolized let‐7b with Kras Vaccine (KVax) can achieve significantly enhanced tumor‐suppressive efficacy.

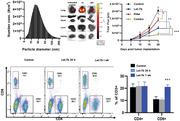

## 1 Introduction

MicroRNAs (miRNAs) are noncoding small RNAs that act as post‐transcriptional repressors and regulators of gene expression. MiRNAs are grossly dysregulated in human cancers, including lung cancer, and they play a critical role in cancer development and progression. Oncogenic miRNAs are typically overexpressed whereas miRNAs that act as tumor suppressors are often underexpressed in cancer cells.^[^ ^1^, ^2^ ^]^ miRNAs are attractive preventive or therapeutic agents for many human health conditions. Their pleiotropic nature makes them particularly attractive for diseases with a multifactorial origin and for which there are no effective treatments. In 2018, the FDA approved the first small‐interfering RNA (siRNA) drug, patisiran, for a rare polyneuropathy caused by hereditary transthyretin‐mediated amyloidosis.^[^ ^3^, ^4^, ^5^ ^]^ In 2019, a second siRNA drug, givosiran, was approved to treat the rare genetic condition acute hepatic porphyria.^[^ ^6^ ^]^ These milestones paved the way for translating additional siRNA and miRNA drugs for clinical use.

The well‐characterized let‐7 miRNA family functions as tumor suppressors; let‐7 genes map to different chromosomal regions that are frequently deleted in lung cancer.^[^ ^2^, ^7^ ^]^ Let‐7 is consistently among the most altered microRNAs in lung cancer tissues, and let‐7 miRNAs negatively regulate multiple oncogenes including RAS, MYC, and HMGA2, as well as cell‐cycle progression regulator genes such as CDC25A, CDK6, and cyclin D2.^[^ ^8^, ^9^, ^10^, ^11^, ^12^ ^]^ A recent study showed that let‐7 miRNAs (let‐7a, let‐7c, or let‐7e) post‐transcriptionally suppress PD‐L1 expression in cancer cells, suggesting that let‐7 miRNAs may promote antitumor immunity in vivo.^[^ ^13^ ^]^ A significant role for the let‐7 miRNA family members (let‐7a, let‐7b, let‐7c, let‐7d, let‐7e, let‐7f, let‐7g, or let‐7i) on the homeostasis and function of CD8 T‐lymphocytes has also been reported,^[^ ^14^ ^]^ indicating that administration of let‐7 miRNAs could potentially modulate host immune responses to developing tumors.

Inhaled medications have been available many years for treating lung diseases such as asthma and chronic obstructive pulmonary disease.^[^ ^15^ ^]^ In comparison to systemic administration, drugs delivered directly to the lungs via inhalation can result in better efficacy at lower doses with decreased toxicity.^[^ ^16^, ^17^, ^18^, ^19^ ^]^ Aerosol delivery of therapeutic drugs for lung cancer in humans has been reported to be an effective route of delivery with little systemic distribution of the therapeutic agents.^[^ ^20^ ^]^ In our previous studies, we successfully used aerosol delivery to reduce side effects associated with systemic administration of drugs, such as liver toxicity.^[^ ^16^, ^17^, ^18^, ^19^ ^]^ Particle size of the target agent is crucial for determining the suitability of aerosol delivery strategies. Smaller particles are more likely to be deposited deep in the airways and lung tissue. Since mice have a much smaller respiratory tract than humans, the optimal particle size for mouse inhalation is less than 0.3 µm. With our collision atomizer, the mass median diameter is less than 0.2 µm, which is favorable for aerosolized drug delivery to mice.

In previous studies, systemic let‐7b administration was able to prevent and treat Kras‐driven lung tumors.^[^ ^21^, ^22^, ^23^ ^]^ The current study is the first investigation of the efficacy of aerosolized let‐7b in lung cancer prevention and found that let‐7b given via aerosol exhibited striking tumor inhibition in both the benzo[*a*]pyrene (B[*a*]P)‐induced and a syngraft model of lung cancer without causing detectable side effects. Using single‐cell RNA sequencing (scRNA‐seq), we found that aerosolized let‐7b treatment decreased the expression of PD‐1 in CD8+ T cells and reduced PD‐L1 expression in lung tumor cells. Let‐7b treatment also significantly changed the percentages of distinct subpopulations of CD8+ tumor‐infiltrating lymphocytes (TILs). The proportion of CD8+ T cells that mediate antitumor functions (effector memory‐like CD8+ TILs) was increased significantly by let‐7b treatment as compared to control. Let‐7b treatment also led to the accumulation of CD8+ T cells, granzyme B+ CD8+ T cells, and IFN‐*γ*+ CD8+ T cells in tumors, and a decrease in intratumoral granulocytic myeloid derived suppressor cells (G‐MDSCs). Together, our results indicate that let‐7b miRNAs promote antitumor immunity in vivo and that treatment with aerosolized let‐7b mimic is a promising approach for lung cancer prevention.

## 2 Results

### 2.1 The Size Distribution of the let‐7b miRNA Aerosols and Tissue Accumulation

**Figure 1:**
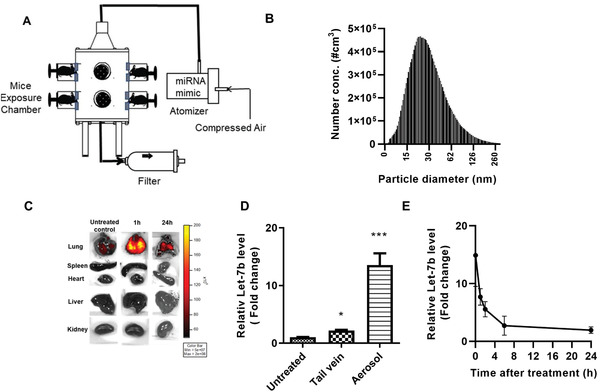
Schematic diagram of aerosol delivery system, size distribution, and let‐7b deposition in lungs. A) Schematic diagram of the aerosol delivery system. B) Size distribution (in nanometers) of let‐7b miRNA mimic particles in the exposure chamber. C) Images showing specific targeting and retention of fluorescent let‐7b‐cy5 in the lungs and other tissues of mice treated with the aerosol form. D) Lung levels of let‐7b miRNA following administration by aerosol or tail vein injection. Data presented as mean ± SEM; *n* = 3; *p < 0.05 and ***p < 0.001. *p*‐values are calculated using Student's *t*‐test. Lungs were removed and were snap frozen in liquid nitrogen. E) Relative levels of let‐7b in the lung over time after a single dose of aerosolized let‐7b miRNA. Mice were euthanized and lung tissues were processed and analyzed for let‐7b miRNA mimic levels at 0, 1, 2, 6, and 24 h after treatment (*n* = 3).

The size distribution of aerosolized let‐7b particles was measured using our custom‐built collision atomizer (**Figure** **1**A). The geometric median diameter (GMD) was 29.0 nm (Figure 1B), the geometric standard deviation (GSD) was 1.9, and the mass median diameter (MMD) was 35 nm. The total concentration was 2.5 µg L^−1^. These particle sizes are suitable for mouse lung inhalation to achieve efficient deposition in mouse bronchioles, terminal bronchioles, and alveoli. Assuming that all the drug particles could completely deposit in the mouse's lung once they were inhaled, the dose of aerosolized let‐7b was estimated as follows^[^ ^24^, ^25^ ^]^

(1) $$\mathrm{Dose}=\frac{C_{\mathrm{aerosol}}\times \mathrm{RMV}\times t}{M_{\mathrm{body}}}$$

where *C*_aerosol_ is the aerosol mass concentration (mg L^−1^), which was measured by the SMPS system, RMV is the respiratory minute volume of the mouse (0.025 L min^−1^, based on Guyton's formula), *t* is the exposure time (10 min), and *M*_body_ is the body weight. Based on the calculation, the deposition dose is 28.4 µg kg^−1^. To evaluate the deposition of let‐7b in the lungs versus other organs, aerosolized let‐7b was administered to mice. Ex vivo images of organs taken 1 and 24 h later showed that the miRNA nanoparticles specifically accumulated in the lungs, with little or no accumulation in other tissues (Figure 1C). We also investigated the accumulation/distribution of neutral lipid emulsion (NLE)‐delivered miRNA in the lungs when given by intravenous tail‐vein injection versus aerosol. Aerosolized let‐7b resulted in significantly higher levels of let‐7b miRNA in the lungs when compared with tail vein delivery (Figure 1D), indicating that aerosol delivery leads to more efficient deposition in the lungs. Pharmacokinetic analysis showed that the highest levels of let‐7b in mouse lungs were present immediately after aerosol delivery, and levels subsequently declined over time (Figure 1E).

### 2.2 The Chemopreventive Efficacy of Aerosolized let‐7b miRNA in Kras‐Driven Mouse Lung Cancer Development

**Figure 2:**
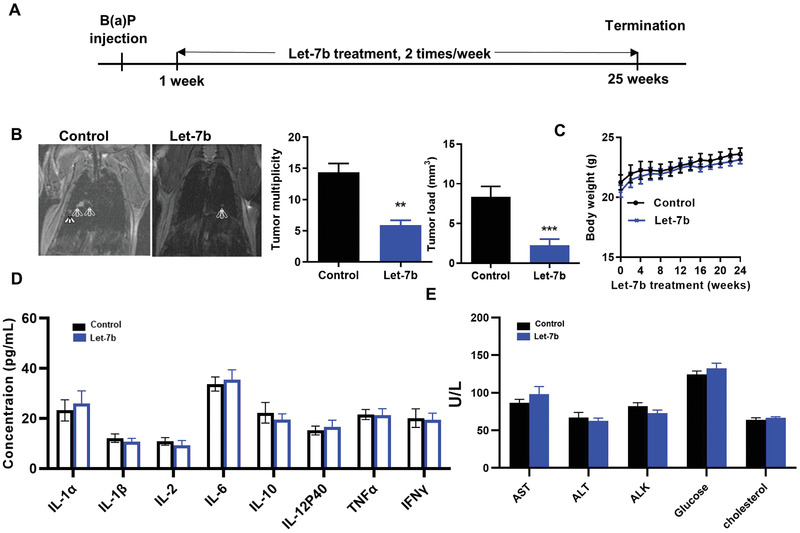
Efficacy of aerosolized let‐7b miRNA in the B(*a*)P‐induced lung cancer model. A) Experimental design. B) Efficacy of let‐7b treatment in decreasing tumor multiplicity (left panel, arrows point to tumors); the bar graphs show effects on tumor multiplicity and tumor load (right panel). Data presented as mean ± SEM; *n* = 10; *p < 0.001 and ***p < 0.001. *p*‐values are calculated using Student's *t*‐test. Potential toxicities of aerosolized Let‐7b were assessed by examining C) body weights, D) serum cytokines, and E) plasma levels of the liver enzymes (*n* = 6).

Efficacy of aerosolized let‐7b in the B[*a*]P‐induced lung cancer model in A/J mice was tested according to the experimental design shown in **Figure** **2**A. Smoke‐induced lung tumor mouse models do not consistently induce lung tumors,^[^ ^26^, ^27^ ^]^ whereas tumors induced by the carcinogen B[*a*]P carry higher mutational loads and contain KRAS activating mutations.^[^ ^28^ ^]^ One week before the experimental endpoint, the tumors were evaluated by magnetic resonance imaging (MRI) imaging of three representative animals per group. MRI imaging demonstrated differences between control and let‐7b mice (Figure 2B, left panel). At the endpoint, the tumor number and load in control mice were 14.4 ± 1.6 and 8.4 ± 1.3 mm^3^, respectively (*p* < 0.001), while tumor multiplicity and load in let‐7b‐treated mice were 5.9 ± 0.8 and 2.3 ± 0.8 mm^3^, respectively (*p* < 0.001) (Figure 2B, right panels). Therefore, let‐7b miRNA treatment induced significant inhibition of lung tumor multiplicity and tumor load. During the 24 weeks of let‐7b administration, no obvious side effects were noted, including no changes in body weight (Figure 2C), no change in serum cytokines (Figure 2D), and no liver toxicity (Figure 2E).

### 2.3 scRNA‐seq Revealed Profound Immune Alterations in the Microenvironment of Let‐7b‐Treated Tumors

**Figure 3:**
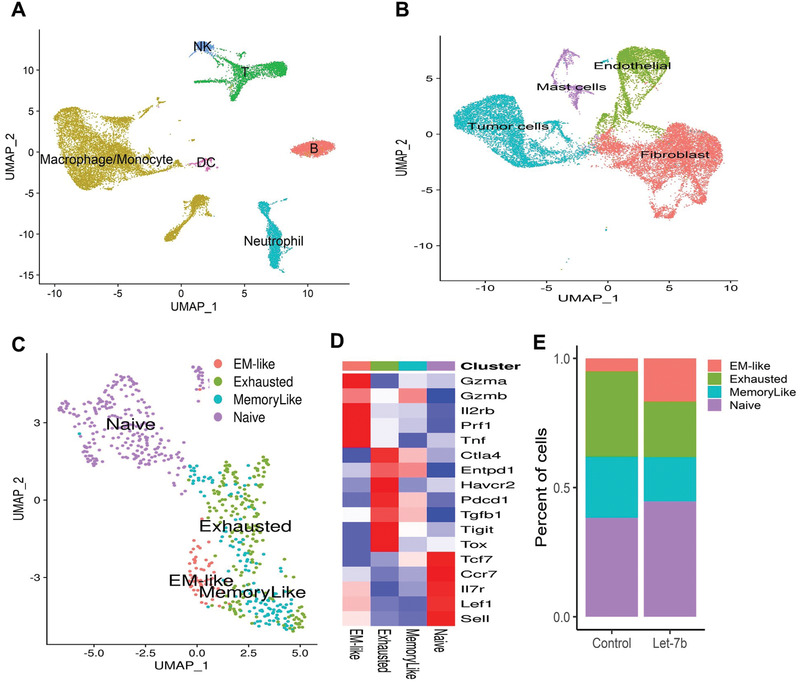
Use of scRNA‐seq to characterize CD8 TIL subsets in lung tumors from A/J mice. A) Clustering of CD45+ immune cells. B) Clustering of CD45− cells including lung tumor cells. C) Clustering of intratumoral CD8+ T cells into the four indicated CD8 TIL subsets. D) The heatmap shows expression of the marker genes for the four tumor‐infiltrating CD8+ T cell states in the mice lung tumor CD8+ TILs. E) Let‐7b treatment increased the proportion of the EM‐like CD8+ TILs and decreased the proportion of exhausted and memory‐like CD8+ T cells in mouse lung (*n* = 2, *p* = 0.04, and *p* = 0.05, respectively).

To better understand the effects of let‐7b on immune function, scRNA‐seq was performed on both tumor cells (CD45−) and immune cells (CD45+) isolated from B[*a*]p‐induced lung tumors in mice from the different treatment groups. The overall clustering of CD45+ and CD45− cells is shown in **Figure** **3**A,B. Unsupervised clustering of CD8+ TILs by using the TILPRED program (https://github.com/carmonalab/TILPRED)^[^ ^29^ ^]^ identified the presence of four CD8+ TIL subsets with distinct transcriptomic profiles (Figure 3C). The CD8 subsets included naïve, effector‐memory (EM)‐like, memory‐like, and exhausted cells. The heatmap of marker genes for these four CD8+ TIL subsets is shown in Figure 3D. The EM‐like (effector memory) CD8+ T cells coexpress cytotoxicity genes (Gzma, Gzmb, and Prf1) and memory genes (Lef1, Sell, and Il7r). EM‐like CD8+ T cells also lack expression of inhibitory receptors (i.e., Pdcd1, Tigit, etc.) and the exhaustion‐related transcription factor Tox. Exhausted CD8+ T cells coexpress inhibitory receptors (Pdcd1 (PD‐1), Ctla4, Entpd1 (CD39), Havcr2 (Tim3)), but lack Tcf7. Memory‐like CD8+ T cells are progenitor exhausted cells that coexpress Pdcd1 and Tcf7 and lack Havcr2. Naive CD8+ T cells have high expression of Tcf7, Lef1, and Il7r, but no expression of cytotoxicity genes or T cell activation markers. Let‐7b treatment significantly altered the percentages of distinct CD8+ TIL subsets in the tumor microenvironment (TME) (Figure 3E). The proportion of CD8+ T cells mediating antitumor function (EM‐like CD8+ TILs) was increased significantly by let‐7b treatment compared to control (*p* = 0.04). In contrast, exhausted CD8+ T cells, which are associated with impaired function of antitumor immune response,^[^ ^29^ ^]^ were decreased by let‐7b. The abundance of progenitor exhausted CD8+ T cells (memory‐like CD8+ T cells) was also decreased by let‐7b treatment. These data suggest that let‐7b improves the overall composition of beneficial antitumor CD8+ TILs.

For the CD45− cell population, we utilized the Seurat R package3 to perform fine clustering of single cells. The numbers of high‐quality CD45− cells available for in‐depth analysis after filtering were 12 563 and 8804 for the Scr (mice treated with scrambled control miRNA) and let‐7b groups (mice treated with let‐7b miRNA), respectively. Gene expression data from single cells of both groups were aligned and projected in a 2D space through uniform manifold approximation and projection (UMAP)^[^ ^30^ ^]^ to allow identification of the cell populations among the CD45− cells. Using the canonical markers for potential cell populations, we identified four major cell clusters of the CD45− cells: malignant cells, fibroblasts, endothelial cells, and mast cells (Figure S1, Supporting Information). After dissecting out the malignant cells from the overall CD45− cells (“Tumor cells” in Figure 3B), we were able to analyze the differentially expressed genes between the let‐7b and scrambled control groups in the “pure” tumor cells. Gene set enrichment analysis (GSEA) revealed that the Kras signaling activated gene set was significantly decreased in let‐7b‐treated tumor cells compared to the Scr control tumor cells (Figure S2A, Supporting Information; normalized enrichment score (NES) value = −2, *p*‐value = 0.004, and false discovery rate (FDR) value = 0.0285). Another cancer signaling pathway, Hedgehog, was also significantly downregulated in the let‐7b‐treated group compared to the Scr control group (Figure S2B, Supporting Information; NES value = −1.7, *p*‐value = 0.035, and FDR value = 0.07). In addition, multiple cell cycle pathways were significantly inhibited in the let‐7b group, including G2/M phase (Figure S2C, Supporting Information; NES value = −2.7, *p*‐value = 0, and FDR value = 5E‐04) and mitotic spindle assembly (Figure S2D, Supporting Information; NES value = −2, *p*‐value = 0.004, and FDR value = 0.02). In contrast, pathways associated with cell death (apoptosis) were significantly upregulated in the let‐7b treatment group (Figure S2E, Supporting Information; NES value = 3.04, *p*‐value = 0, and FDR value = 0).

### 2.4 Let‐7b Increased the Effector‐Memory Function of T Cells and Reduced G‐MDSCs and Tregs in Lung Tumors

**Figure 4:**
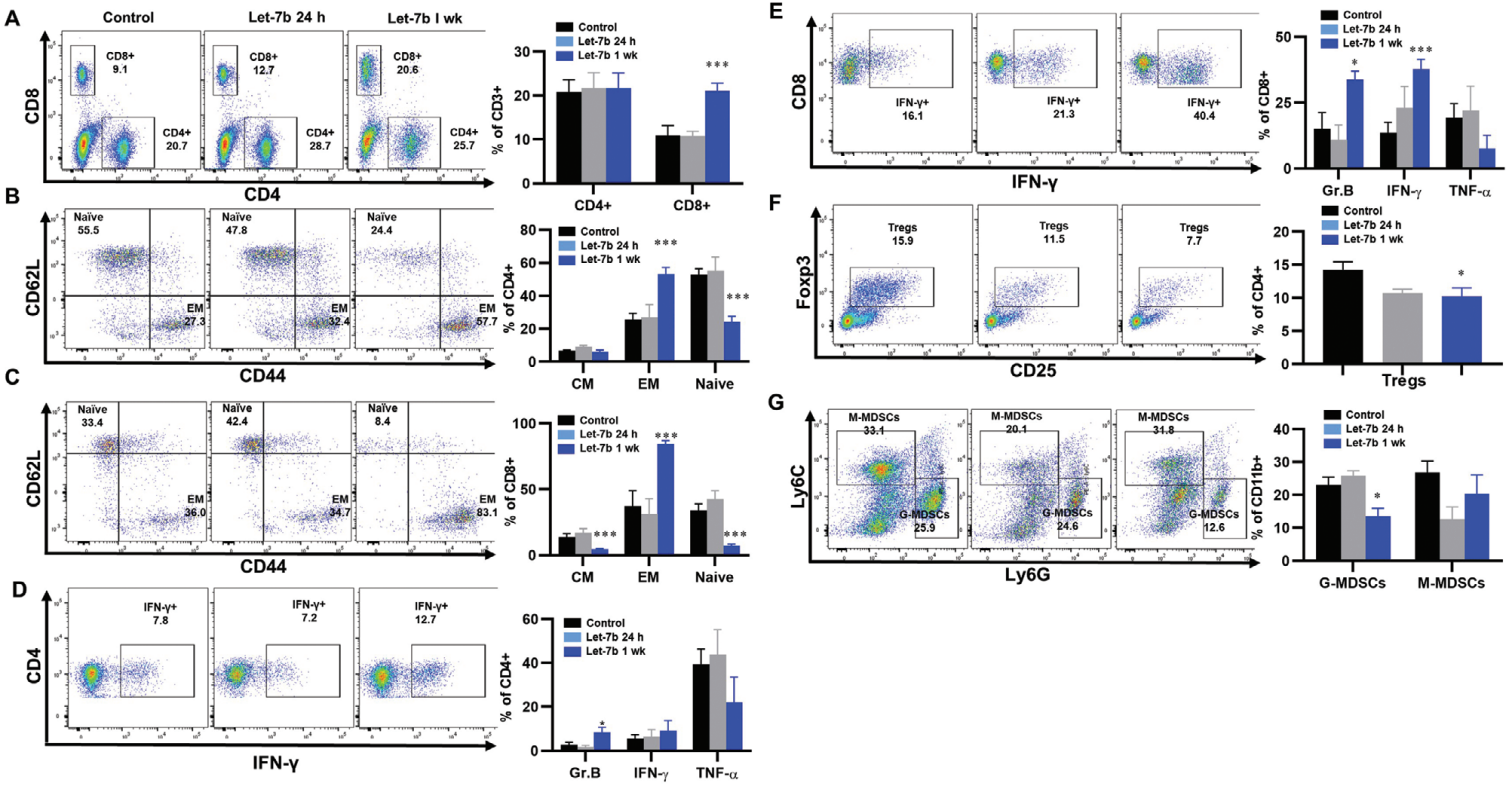
The effects of let‐7b on the tumor microenvironment. A) The effect of let‐7b on tumor infiltrating CD8+ T cells; B,C) the effect of let‐7b on tumor infiltrating effector memory CD4+/CD8+ T cells and naïve CD4+/CD8+ T cells; D,E) the effect of let‐7b on granzyme B and IFN‐*γ* secretion; F,G) the effect of let‐7b on Tregs and GMDSC. Data presented as mean ± SEM; *n* = 6; *p < 0.05 and ***p < 0.001. *p*‐values are calculated using one‐way ANOVA analysis.

To validate our scRNA‐seq findings, mice with Kras‐driven lung tumors (LKR13) were treated with let‐7b and TILs analyzed by flow cytometry for expression of specific cell surface markers. In tumor‐bearing mice one‐week post‐treatment, let‐7b led to increased accumulation of CD8+ T cells *(p* < 0.001) (**Figure** **4**A), increased effector‐memory CD4+ and CD8+ T cells (*p* < 0.001), and decreased naïve CD4+ and CD8+T cells (*p* < 0.001) (Figure 4B,C). Let‐7b treatment also increased granzyme B+ CD8 T cells (*p* < 0.05) and IFN‐*γ*+ CD8 T cells (*p* < 0.001) in Kras mutant lung tumors (Figure 4D,E), and decreased intratumoral Tregs (*p* < 0.05) and G‐MDSC cells (*p* < 0.05) (Figure 4F,G).

### 2.5 The Immune‐Related Role of let‐7b in Inhibiting Lung Tumor Development

**Figure 5:**
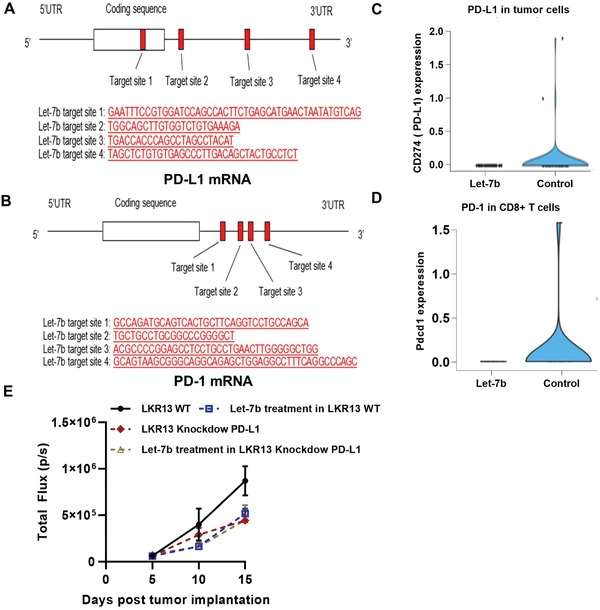
Suppression of PD‐1 and PD‐L1 expression in let‐7b‐treated mice. Predicted binding sites for let‐7b miRNA in human A) PD‐L1 mRNA and B) PD‐1 mRNA. scRNA‐seq was used to assess C) expression of PD‐L1 in CD45− tumor cells from let‐7b‐treated mice, and D) expression of PD‐1 in CD45+ immune cells from let‐7b‐treated mice. E) Efficacy of let‐7b in parental LKR13 cells or LKR13 shPD‐L1 KD cells. Data presented as mean ± SEM; *n* = 6; *p < 0.05 and ***p < 0.001. *p*‐values are calculated using one‐way ANOVA analysis.

The binding sites for let‐7b in human PD‐L1 mRNA (**Figure** **5**A) and PD‐1 mRNA (Figure 5B) were identified by miRwalk software. scRNA‐seq data showed that let‐7b treatment reduced PD‐L1 expression in lung tumor cells (Figure 5C) and decreased the expression of PD‐1 in CD8+ T cells (Figure 5D). These data suggest that let‐7b can inhibit PD‐1/PD‐L1 interactions by blocking expression of both molecules in the TME. We hypothesized that in vivo tumor‐suppressive efficacy of let‐7b is mediated, at least in part, by immune‐promoting effects such as downregulation of PD‐L1 in tumor cells. To test this hypothesis, we used shPD‐L1 lentivirus to knockdown PD‐L1 in LKR13 cells (PD‐L1 KD LKR13 cells). Aerosolized let‐7b was given after the mice were inoculated with these tumor cells, and tumor growth was monitored by bioluminescence imaging (BLI). In LKR13 wild‐type (WT)‐inoculated mice, let‐7b treatment inhibited tumor burden and in PD‐L1 knockdown LKR13‐inoculated mice, effect of let‐7b was mostly abrogated with no significant further inhibition was observed (Figure 5E). Together with the scRNA‐seq data, these data suggest that the antitumor effects of let‐7b, at least in part, depend on PD‐L1.

### 2.6 The Efficacy of a Kras Vaccine (KVax) in Combination with Aerosolized let‐7b miRNA on LKR13 Syngraft Tumors

**Figure 6:**
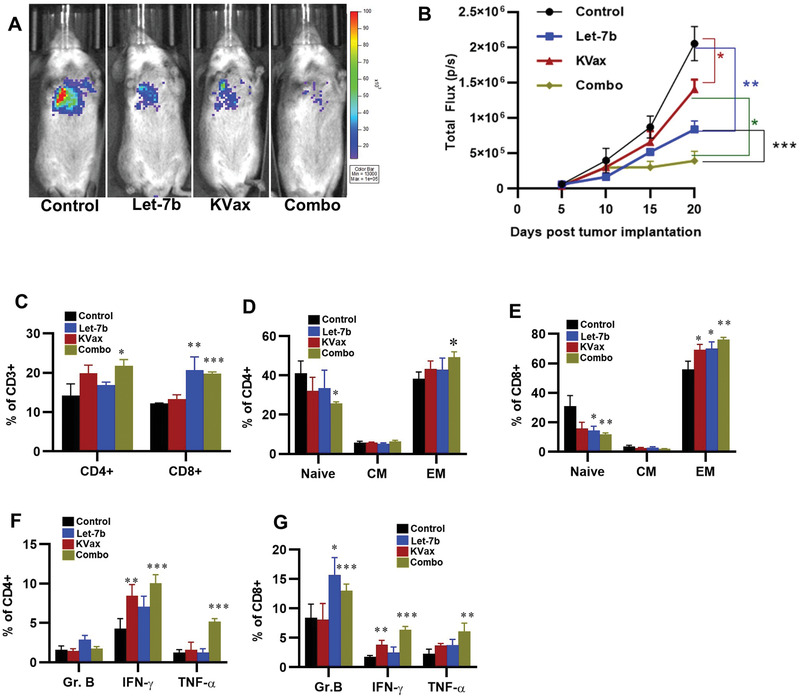
Efficacy of a KVax in combination with aerosolized let‐7b miRNA on LKR13 syngraft tumors. A) Representative bioluminescence images from mice bearing lung tumors treated with control, KVax, Let‐7b, or the combination (Combo). B) Quantitative data for the bioluminescence imaging. Data presented as mean ± SEM; *n* = 6; *p < 0.05 and ***p < 0.001. *p*‐values are calculated using one‐way ANOVA analysis. C–F) Effects of let‐7b treatment on the tumor microenvironment in this syngraft model. C) The effect of treatment in tumor infiltrating CD8+ T cells. D,E) The effect of treatment in EM‐like CD8+ TILs and CM‐like TILs. F,G) The effect of treatment in granzyme B, IFN‐*γ*, and TNF‐*α*.

Since let‐7 miRNA inhibits the PD‐1/PD‐L1 pathway in the TME, we hypothesized that combining a Kras MHCII‐restricted, multipeptide vaccine (KVax) (which promotes CD4+ T helper type 1 immune responses)^[^ ^31^, ^32^ ^]^ and aerosolized let‐7b miRNA would enhance type I adaptive immunity and improve antitumor efficacy. KVax has been shown to be highly effective in a doxycycline‐inducible KRAS^G12D^ lung cancer prevention model. This vaccine mostly induces expansion of CD4^+^ T cells in the spleen and lymph nodes and elicits robust Kras‐specific T cell responses that inhibit tumor growth.^[^ ^31^, ^32^ ^]^ To test the ability of let‐7b to potentiate the efficacy of KVax, we established lung tumors via tail vein injection of LKR13 cells. Three days after tumor inoculation, two weekly KVax vaccinations resulted in ≈30% inhibition of tumor growth (*p* < 0.05), whereas let‐7b treatment alone decreased tumor growth by ≈60% (**Figure** **6**A,B) (*p* < 0.001). The combined treatment enhanced the overall efficacy to ≈80% inhibition of tumor growth (*p* < 0.001). Further analysis of TILs harvested from each treatment group showed that while KVax treatment boosted CD4+ T cell infiltrates in the TME, let‐7b mostly enhanced CD8+ T cell frequencies; the combined treatment led to the accumulation of both CD4+ and CD8+ T cells in the TME (Figure 6C). Increases in frequencies of effector‐memory T cells were also observed (Figure 6D,E). Not only did the percentages of these T cell populations increase, but function of these T cells was also significantly boosted as reflected by increased levels of granzyme B+ and IFN‐*γ*+ T cells (Figure 6F,G).^[^ ^2^, ^8^, ^9^, ^11^, ^12^ ^]^

## 3 Discussion

The let‐7 miRNA family functions as tumor suppressors in lung cancer.^[^ ^1^, ^2^, ^3^, ^13^ ^]^ After comparing lung adenocarcinoma tumor samples with control normal lung tissues from TCGA project, we found that let‐7b expression is significantly lower in lung cancer tissue than in the adjacent normal tissues, and low let‐7b expression is associated with a shorter overall survival than those with high let‐7b (Figure S3, Supporting Information). While the role of let‐7 in suppressing tumor development and target oncogenes, and regulating the cell cycle, cell signaling, and maintenance of differentiation are known,^[^ ^13^, ^33^, ^34^, ^35^ ^]^ the immune regulatory role(s) of let‐7b in lung tumorigenesis has not been well‐defined. Recent reports indicate that Let‐7 miRNAs post‐transcriptionally suppress PD‐L1 and PD‐1 expression in the TME, suggesting that let‐7 miRNAs may promote antitumor immunity in vivo.^[^ ^13^, ^35^ ^]^ We therefore investigated if aerosolized let‐7b miRNA could effectively block mouse lung cancer development and progression and exhibit a favorable safety profile. We also examined the tumor‐intrinsic versus immune‐associated roles of let‐7b miRNA in lung tumorigenesis.

Let‐7b significantly inhibited lung cancer in a B[*a*]P‐induced model with no detectable toxicities. scRNA‐seq was conducted on B[*a*]P‐induced primary lung tumors treated with let‐7b miRNA. In our analysis of tumor cells (CD45‐ cell population), mechanisms underlying the antitumor efficacy of let‐7b treatment for lung cancer appear to be related to inhibition of important cancer signaling pathways including Kras and Hedgehog. Moreover, let‐7b treatment significantly inhibits different cell cycle phases in tumor cells and promotes apoptosis of lung tumor cells, which, confirms that let‐7b functions as tumor suppressor.^[^ ^1^, ^12^ ^]^

In addition to its impact on regulation of signaling pathways in tumor cells, let‐7b treatment significantly shifted the percentages of distinct CD8+ TIL subsets toward antitumor immunity. EM‐like CD8+ TILs were significantly increased, and exhausted CD8+ TILs were significantly decreased by let‐7b. Flow cytometry analysis of tumors confirmed these scRNA‐seq findings in that let‐7b treatment not only enhanced CD8+ TILs within tumors, but also potentiated their cytotoxic function as reflected in increased secretion of cytokines such as IFN‐*γ*. There could be multiple mechanisms by which let‐7b promotes CD8+ T cell accumulation in tumor tissues. One reason could be the decreased presence of myeloid‐derived suppressor cells (MDSCs).^[^ ^36^ ^]^ Previous studies suggest that tumor‐derived CCL2 recruits CCR2+ MDSCs, which in turn impedes the infiltration of CD8+ T cells.^[^ ^37^ ^]^ Our flow cytometry analysis supports this mechanism, suggesting that over time, intermittent aerosolized delivery of let‐7b reduces the number of suppressive myeloid cells (G‐MDSCs) as well as Tregs within tumors. Another mechanism by which let‐7b induces the proliferation and effector function of tumor‐specific CD8+ T cells is via targeting PD‐1/PD‐L1. Our scRNA‐seq analysis suggests that let‐7b can effectively inhibit PD‐1/PD‐L1 interactions by blocking expression of both molecules in the TME. Our in vivo results also show that let‐7b stimulates T cell‐mediated antitumor activity.

Our MHC II‐restricted KVax is highly effective in preventing Kras‐driven lung tumor development in multiple models.^[^ ^31^, ^32^ ^]^ While MHC I‐restricted epitopes are specific to class I (HLA‐I) molecules, MHC II‐restricted epitopes developed by the previously reported algorithm^[^ ^7^, ^33^, ^34^ ^]^ are promiscuous MHC class II‐binders and thus can bind to multiple HLA‐DR alleles, increasing the applicability to broader populations of at‐risk individuals and cancer patients.^[^ ^38^, ^39^ ^]^ KVax was designed to be MHC II‐restricted with the goal of specifically inducing the activation and expansion of CD4+ T cells, and its peptides are 100% identical to human mutant KRAS. In the studies reported here, combining KVax with let‐7b miRNA enhanced cytotoxic CD8+ TILs in the TME.^[^ ^40^ ^]^ By evaluating TILs from tumors treated with the combination of KVax and let‐7b, we indeed observed that this combination significantly increases tumor infiltration by both CD4^+^ and effector CD8^+^ T cells, and simultaneously enhances the function of these T cells as reflected in enhanced cytokine production (IFN‐*γ* and granzyme B).

In summary, our experiments demonstrate that aerosolized let‐7b inhibits lung tumor growth and progression via both tumor‐intrinsic and immune‐mediated mechanisms. Let‐7b promotes CD8+ T cell accumulation in tumor tissues, and the effector function of tumor‐specific CD8+ T cells is enhanced through targeting the PD‐1/PD‐L1 pathway. Our data support advancing the use of aerosolized let‐7b for lung cancer prevention and/or treatment, and the data indicate that combining let‐7b with KVax can achieve even better tumor‐suppressive efficacy. This combination could represent an exciting new approach for the immunoprevention of lung cancer.

## 4 Experimental Section

### Reagents

Benzo[*a*]pyrene (B[*a*]P) and tricaprylin were purchased from Sigma Chemical Co. (St. Louis, MO). NLE was purchased from BIOO Scientific (Austin, TX). The let‐7b mimic was purchased from Dharmacon. Using proprietary technology, the mimic was chemically enhanced to preferentially program RNA‐induced silencing complex (RISC) with an active miRNA sequence (https://horizondiscovery.com/‐/media/Files/Horizon/resources/Product‐inserts/miridian‐mimics‐inhibit‐neg‐contr‐prodinsert.pdf). Fluorescent let‐7b‐cy5 used in ex vivo imaging studies was obtained from Dharmacon. The miRNeasy mini kit, miScript II RT Kit, miScriptSyBr Green PCR Kit, hsa‐let‐7b primer, and small nuclear RNA U6 were purchased from Qiagen (Valencia, CA). Chromium Single Cell 3′ v3 Reagent Kits (10x Genomics) and NextSeq 500/550 High Output sequencing reagent Kits v2 (150 cycles) (Illumina) were used according to the manufacturer's protocol.

### Animals and Cell Lines

A/J mice, FVB mice, and SV129 mice were purchased from Jackson Laboratory. LKR13 cells, which originated from KrasLA1 mice, were a generous gift from Dr. Jonathan M. Kurie (MD Anderson). RPMI‐1640 medium used to grow LKR13 cells was supplemented with 10% fetal bovine serum (FBS, Sigma) and 1% penicillin and streptomycin (Thermo Scientific). Cells were cultured at 37 °C in a 5% CO_2_ incubator. To establish LKR13‐Luciferase (LUC) expressing cells, LV‐CMV‐Puromycin‐firefly luciferase (Kerafast) was transduced into LKR13 cells according to the manufacturer's protocol. 1 × 10^5^ cells of each line were plated in 6‐well plates, and 24 h later the medium was replaced with transduction medium containing lentivirus that expresses the puromycin luciferase fusion protein and polybrene (8 µg mL^−1^). 48 h after transduction, the infected cells were selected with puromycin (2 µg mL^−1^) for 3 days; the pooled cells stably expressing luciferase were used in the in vivo experiments. All procedures were in accordance with the Medical College of Wisconsin Institutional Animal Care and Use Committee.

### Aerosol Procedure

The let‐7b miRNA mimic was atomized into droplets using the custom‐made collision type atomizer. The total exposure time of mice to the let‐7b miRNA was 10 min per treatment, twice per week. These inhalation exposures were given using a custom‐built nose‐only exposure chamber. The effluent aerosol was discharged from an opening at the bottom of the chamber. Mice were exposed one at a time to the aerosol by placing their noses into the cone of the apparatus. The diameter of particles was determined to be in the nano–millimeter range, which is favorable for mouse inhalation. The size distribution of drug aerosol produced by the atomizer was determined by a scanning mobility particle sizer (SMPS) spectrometer including a bipolar aerosol charger, a differential mobility analyzer (DMA, TSI model 3081) with the DMA platform (TSI model 3080), and an ultrafine condensation particle counter (UCPC, TSI model 3025). GMD, MMD, GSD, and particle concentration of dry particle size distributions were obtained from the measurements.^[^ ^18^ ^]^

### Biodistribution and Pharmacokinetics of the let‐7b miRNA

For all aerosol treatments, an NLE was used to deliver let‐7b miRNA. NLE consists of 1,2‐dioleoyl‐sn‐glycero‐3‐phosphocholine, squalene oil, polysorbate 20, and an antioxidant that, in complex with synthetic miRNAs, forms nanoparticles in the nanometer diameter range. For the biodistribution study of let‐7b by tail vein injection or by aerosol, eight‐week‐old FVB mice were separated into 3 groups: 1) untreated controls; 2) tail vein injection; 3) aerosolized delivery (*n* = 3 each). Mice from group 2 were given a single intravenous tail‐vein injection of 20 µg let‐7b miRNA formulated with 200 µL NLE according to the manufacturer's instructions. Mice from group 3 were given aerosolized let‐7b, which was also formulated with NLE. 10 min after the dose was administered, the mice were euthanized by CO_2_ asphyxiation to assess the distribution of let‐7b. To study pharmacokinetics of the let‐7b miRNA mimic in mice, eight‐week‐old FVB mice were treated once with aerosolized let‐7b miRNA, and mice were euthanized at 0, 1, 2, 6, and 24 h after treatment. Approximately 25 mg of lung tissue from each mouse was used for analysis. The miRNA was extracted from lungs using the miRNeasy kit (QIAGEN, Valencia, CA) according to manufacturer's guidelines. Briefly, lung tissues were harvested and snap frozen, and the tissue was processed using a tissue homogenizer. Chloroform was then added and mixed thoroughly, followed by centrifugation for 15 min at 12 000 rpm, 4 °C. The aqueous layer was removed, mixed with a 1.5‐fold volume of ethanol, and transferred to a miRNeasy spin column for RNA purification. 1 µg total RNA was transcribed to cDNA using the miScript reverse transcription II kit (QIAGEN, Valencia, CA) in a total volume of 20 µL following the manufacturer's protocol. miRNA qRT‐PCR reactions were performed using miScriptSyBr green PCR kit (QIAGEN) with primers for hsa‐let‐7b; a primer for small nuclear RNA U6 was used as an endogenous control (QIAGEN). Negative controls without template were included in each plate. All qPCR was run in a CFX Connect real time PCR system (Bio‐Rad, Hercules, CA) using the following cycling conditions: 95 °C for 15 min; 40 cycles of denaturation at 94 °C for 15 s, annealing at 55 °C for 30 s, and extension at 70 °C for 30 s with a ramp rate of 1 °C s^−1^.

### Efficacy of let‐7b in the B(*a*)P‐Induced Lung Cancer Model

To characterize the efficacy of let‐7b miRNA on suppressing lung carcinogenesis, the B[*a*]P‐induced lung tumor model in A/J mice was used. Six‐week‐old female A/J mice were injected with the chemical inducer B[*a*]P (single i.p. dose, 100 mg kg^−1^ in 0.2 mL tricaprylin). One week after the B[*a*]P dose, mice were randomized into two groups: 1) scramble miRNA (Scr) control group; 2) let‐7b miRNA group. Mice were treated by aerosol in NLE two times per week. After 24 weeks of the miRNA treatment, mice were euthanized. Lungs were fixed and evaluated under a dissecting microscope to obtain surface tumor count and individual tumor diameter. Tumor volume was calculated based on the following formula: *V* = 4*πr* ^3^/3.^[^ ^18^ ^]^ The total tumor volume in each mouse was calculated from the sum of all tumors. Tumor load was determined by averaging the total tumor volume of each mouse in each group. Body weights were measured weekly. After 22 weeks of treatment, serum was collected and analyzed by Marshfield Labs for cholesterol, glucose, and the liver function enzymes alanine transaminase (ALT), aspartate aminotransferase (AST), and alkaline phosphatase (ALK). Mouse cytokine array/chemokine array 31‐Plex (MD31) kits were used for cytokine analysis (Eve's Technologies); it analyzes a panel of 31 cytokine. Mice serum from control groups or let‐7b‐treated group were collected and sent to EVE's Technologies for analysis.

### MRI

A/J Mice were imaged using a 9.4T MRI system (Bruker, Billerica, MA) with a custom birdcage style quadrature coil (Doty Scientific, Columbia, SC). Mice were anesthetized for 2 min initially in a chamber using a mixture of oxygen (3 L min^−1^) and isoflurane (4%). Once an animal was fully anesthetized, ocular lubricant was placed on each eye to prevent drying. The mouse was then moved to the MRI magnet room and placed in a supine position on a plastic bed fitted with a nose cone that continuously supplied oxygen (1.4 L min^−1^) and isoflurane (2.5%). Initially, anesthesia was with 2.5% isoflurane and then maintained at 1.5–2.0% isoflurane. The heart rate, body temperature, and respiratory rate were continuously monitored throughout imaging. Both respiratory and cardiac gating with an electrocardiogram were used to ensure that images were consistently acquired during latent periods of the respiratory cycle and at a consistent point during the cardiac cycle. A temperature probe was placed under the mouse to monitor the skin temperature. The mouse was heated to a skin temperature of 37 °C to maintain a core temperature of ≈37.5 °C by blowing thermostatically controlled warm air into the magnet's bore. Tumors were imaged using a multislice multiecho acquisition (MSME). Images were acquired using the following parameters: echo time (TE) = 8.07 ms, repetition time (TR) ≥ 400 ms (variable), matrix = 128 × 128, 1 average, 20 axial slices. The images were analyzed by microDicom software.

### scRNA‐seq Analysis of Mouse Lung Tumors

For scRNA‐seq, B[*a*]P‐induced primary lung tumors were harvested and pooled from each mouse at the end of the study, then minced into 1–2 mm^3^ pieces, and digested at 37 °C for 20 min with mouse tumor dissociation buffer (Miltenyi Biotec, CA) to generate single‐cell suspensions. Red blood cells were lysed with ammonium–chloride–potassium (ACK) buffer, single‐cell suspensions were stained with 7‐AAD and CD45 on ice for 30 min, and CD45− and CD45+ populations were sorted by flow cytometry. For single‐cell library preparation, flow‐sorted CD45− or CD45+ cells were pelleted by centrifugation at 300 × *g* for 5 min and counted manually using a Neubauer Chamber. Approximately 1.6 × 10^4^ cells were loaded onto the 10X Chromium controller as per the manufacturer's instructions. The scRNA‐seq libraries were generated by Chromium single cell 3′ v3 reagent Kits (10x Genomics) and sequenced using NextSeq 500/550 high output kits v2 (150 cycles) (Illumina) according to the manufacturers’ protocols.

### scRNA‐seq Data Analysis

Raw sequencing data were demultiplexed and converted to gene‐barcode matrices using the Cell Ranger (version 2.2.0) mkfastq and count functions, respectively (10x Genomics). The mouse reference genome mm10 was used for alignment. Data were further analyzed in R (version 3.4.0) using Seurat (version 3). The number of genes detected per cell, the number of unique molecular identifiers (UMIs), and the percent of mitochondrial genes were plotted, and outliers were removed (cells that expressed less than 200 and more than 2500 genes) to filter out doublets (two single cells) and dead cells. Differences in the number of UMIs and percent of mitochondrial reads were regressed out. Raw UMI counts were normalized, and log transformed. To analyze the sequenced CD45− cells from mouse lung tumors, the Seurat R package3 was utilized to perform fine clustering of the single cells.^[^ ^41^, ^42^ ^]^ The gene expression data from all single cells were aligned and projected in a 2D space through UMAP to allow identification of the cell populations among the CD45− or CD45+ cells.

Differential gene expression analysis of scRNA‐seq data was performed as follows: Before differential expression analysis, the computational imputation of zero values was performed to correct for the influence of dropout events (i.e., failure in detecting expressed genes due to low sequencing depth of single cells). Computational methods described previously^[^ ^43^ ^]^ were utilized to perform imputation and other data processing procedures. Specifically, gene expression levels were quantified using metric log2 (TPM+1). Transcripts per million (TPM) is a normalization method for RNA‐seq and should be read as “for every 1 000 000 RNA molecules in the RNA‐seq sample, *x* came from this gene/transcript.” Missing gene expression values were imputed using the scImpute algorithm with default parameters and TPM values and gene lengths (for a gene associated with multiple transcripts, the length of the longest transcript was used) as the input. Imputation was only applied to genes with dropout rates (i.e., the fraction of cells in which the corresponding gene has zero expression value) larger than 50% to avoid overimputation. The imputated scRNA‐seq data were then subjected to differential expression analysis using the DEsingle program to assess differences between the let‐7b and scrambled RNA treatment groups. A list of the overall differential expression results was used as input into the GSEAPreranked tool implemented in the GSEA program. Hallmark gene sets listed in the MSigDB (molecular signatures database: https://www.gsea‐msigdb.org/gsea/msigdb/index.jsp) were used to test gene expression signatures to detect the important biological processes that are affected by let‐7b treatment in the mouse lung cancer model. These analyses may provide insights into the mechanism(s) underlying the efficacy of let‐7b therapy in preventing or treating lung cancer.

### Binding Site Analysis

miRWalk software^[^ ^44^, ^45^, ^46^ ^]^ was used to predict binding sites for *let‐7b* in PD‐L1 and PD‐1 mRNA. To filter the candidate binding sites for let‐7b, the stringent criteria of 1) binding probability >0.9 and 2) free energy <−15 (kJ mol^−1^) was used.

### Efficacy of let‐7b Mimic in the SV129 Mouse Syngraft LKR13‐LUC Model

LKR13 cells stably expressing luciferase were used for establishing syngeneic tumor grafts in vivo. The cells were trypsinized, washed with PBS, and then suspended in PBS at a concentration of 2.5 × 10^6^ cells mL^−1^. A total of 0.2 mL cell suspension was injected into the tail vein of six‐week‐old female SV129 mice. First, the immune effect of short‐term aerosolized let‐7b treatment was examined. Three days after injection of LKR13‐LUC cells, mice were divided into 3 groups (5 mice per group): 1) scramble miRNA (Scr) control tumor control group; 2) tumor with let‐7b treatment group (24 h); 3) tumor with let‐7b treatment group (1 week). Lung tumors were harvested from each mouse at the indicated times and examined by flow cytometry analysis. An MHC II‐restricted multipeptide vaccine that targets Kras WT regions and results in >80% inhibition of Kras‐driven lung tumorigenesis with no overt toxicity was previously developed.^[^ ^31^, ^32^ ^]^

To test the combination of let‐7b with this KVax in the LKR13 syngraft model. LKR13‐LUC cells were inoculated via tail vein injection, and animals were randomized to 4 groups after three days (five mice per group) and treatments with aerosolized let‐7b or KVAX were initiated immediately: 1) control group; 2) KVax injection group; 3) aerosolized let‐7b group; 4) aerosolized let‐7b + KVax group. KVax was given twice subcutaneously, one week apart. Aerosolized let‐7b miRNA was given twice per week. Tumor growth was monitored with the Lumina IVIS‐100 in vivo imaging system (Xenogen Corporation) using d‐luciferin (Xenogen Corporation) as the substrate. Regions of interest (ROI) were created and measured as area flux, defined by radiance (photons per second per square centimeter per steradian).

To test the efficacy of aerosolized let‐7b treatment in tumors with reduced levels of PD‐L1 expression, PD‐L1 was knocked down (KD) in LKR13 cells by shRNA lentivirus (Santa Cruz Biotech). LKR13 cells or PD‐L1 KD LKR13 cells (5 × 10^5^) were injected into the tail vein of six‐week‐old female SV129 mice. Three days after injection of these cells, treatments commenced: 1) control group; 2) let‐7b treatment group; 3) shPD‐L1 control group; 4) shPD‐L1 with let‐7b treatment group. Aerosolized let‐7b miRNA was given twice per week. Mice were imaged using the Lumina IVIS‐100 in vivo Imaging System (Xenogen Corporation). Regions of interest were created and measured as area flux, defined by radiance (photons per second per square centimeter per steradian).

### Flow Cytometry

For immune profiling, tumors were harvested and pooled from each mouse at the end of the study, minced into 1–2 mm^3^ pieces, and digested at 37 °C for 20 min with mouse tumor dissociation buffer (MiltenyiBiotec, CA) as per the manufacturer's instructions to generate single‐cell suspensions. Tumor‐infiltrating leukocytes were directly stained for flow cytometry sorting or analysis. Cells were stained with the following cell surface and viability markers: 7AAD for live/dead cells, BV786 anti‐CD45, APC eFluro780 anti‐CD3, FITC anti‐CD4, BUV396 anti‐CD8a, SB600 anti‐CD19, PE‐Cy7 anti‐CD44, APC anti‐CD62L, and PE anti‐CD25 antibodies. For intracellular cytokine staining, cells were stimulated for 4 h in RPMI medium containing Kras peptides, 10% FBS, 2 × 10^−3^ m l‐glutamine, 50 × 10^−6^ m 2‐mercaptoethanol, 1% penicillin–streptomycin, 1× monensin, and 1× Brefeldin A (Thermofisher Sci). For Foxp3 staining, cells were washed, fixed, permeabilized, and stained with Foxp3/transcription factor eFluor450 staining buffer sets (ThermoFisher) following the manufacturer's instructions. For intracellular cytokine analysis, cells were fixed with 2% paraformaldehyde, permeabilized with 0.5% saponin, and stained with intracellular cytokine staining buffer containing Brefeldin A, APC anti‐granzyme B, PE anti‐IFN‐*γ*, and PE‐Cy7 anti‐TNF‐*α* antibody, and then analyzed by flow cytometry. T cells stained with isotype control antibody were used as negative controls. To detect MDSCs in tumors, cells were stained with PerCP‐Cy5.5 anti‐CD45, FITC anti‐CD11b, APC anti‐CD11c, PE anti‐Ly6G, and PE‐Cy7 anti‐Ly6C Ab. Flow cytometry was conducted using an LSR Fortessa X‐20 or LSR‐II flow cytometer (Becton Dickinson). Data were analyzed using FlowJo software.

### Statistical Analysis

All data were presented as means ± standard error of means (SEM). Statistical analysis was performed using GraphPad Prism Software. To determine which specific groups differed from each other, Tukey's “post‐hoc” test was used. For comparison between 2 groups, paired Student's *t*‐test was performed. For multiple groups (3 groups and above) comparison, one‐way ANOVA analysis was employed with Bonferroni's post‐test. Sample sizes (*n*) were mentioned on each figure legend. *p < 0.05 was considered statistically significant.

## Conflict of Interest

The authors declare no conflict of interest.

## Supporting information

Supporting Information

## Data Availability

Data available on request from the authors.
