# Supplementary material for: Pulmonary Aerosol Delivery of Let‐7b microRNA Confers a Striking Inhibitory Effect on Lung Carcinogenesis through Targeting the Tumor Immune Microenvironment
Source: Adv Sci (Weinh). 2021 Jul 8;8(17):2100629. doi: 10.1002/advs.202100629 (PMC8425922; doi:10.1002/advs.202100629)
Supplement: Supplementary file 1 — Supporting Information [file ADVS-8-2100629-s001.pdf]

© 2021 Wiley-VCH GmbH

ADVANCED SCIENCE
Open Access

Supporting Information

for *Adv. Sci.,* DOI: 10.1002/advs.202100629

Pulmonary Aerosol Delivery of Let-7b microRNA Confers a Striking Inhibitory Effect on Lung Carcinogenesis through Targeting the Tumor Immune Microenvironment

Qi Zhang[1,2#], Jing Pan[1,2#], Donghai Xiong[1,2], Yian Wang[1,2], Mark Steven Miller[3], Shizuko Sei[3], Robert H Shoemaker[3], Alberto Izzotti[4,5] and Ming You[1,2*]

Supplemental figure S1

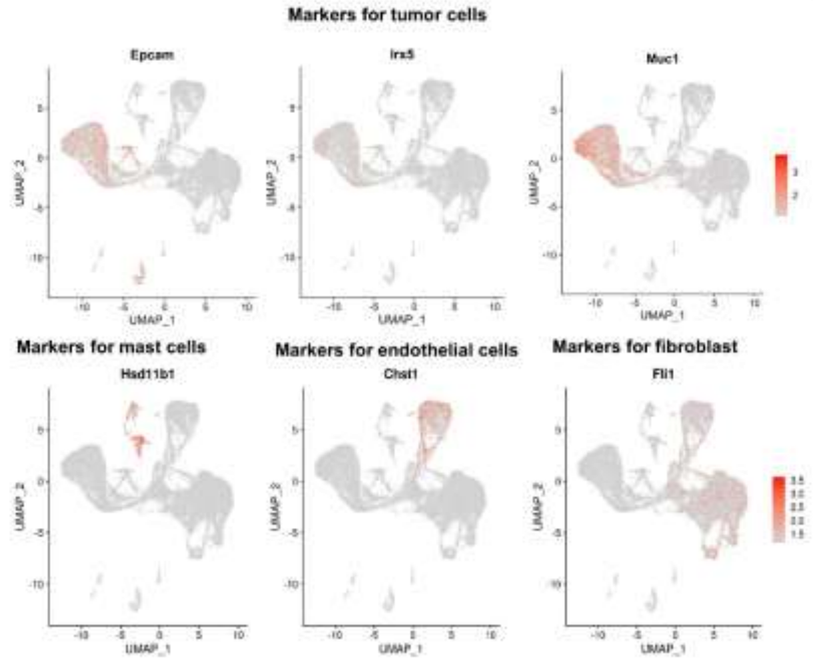

Expression of marker genes for each cell clusters of Cd45- flow sorted single cells

Supplemental figureS2

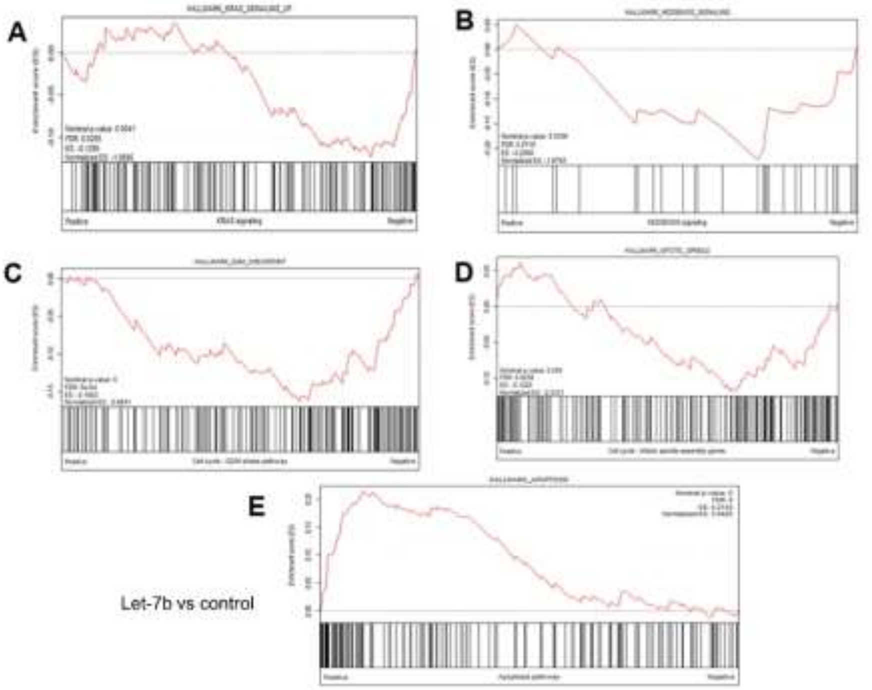

Pathway analysis of scRNA-seq data comparing let-7b treatment to control group.

Supplemental figure S3

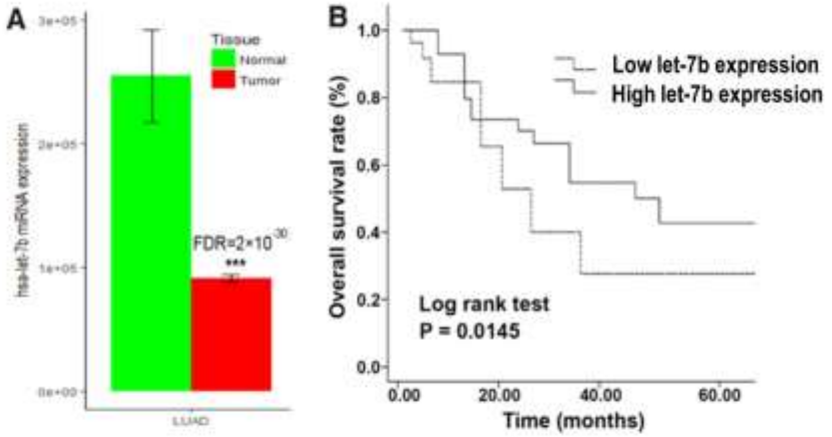

Human let-7b expression was decreased in the lung adenocarcinoma tumor samples compared to the control normal lung tissues from TCGA project. The low expression of let-7b was also associated with worse overall survival outcome of lung cancer patients.

Supplemental figure S4

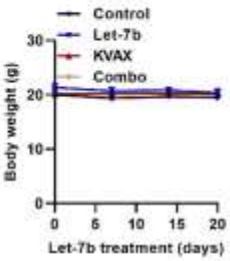

Body weight changes of aerosolized let-7b miRNA or KVAX in the LKR13 lung cancer model
